# Contribution of Embodiment to Solving the Riddle of Infantile Amnesia

**DOI:** 10.3389/fpsyg.2016.00010

**Published:** 2016-01-25

**Authors:** Arthur M. Glenberg, Justin Hayes

**Affiliations:** ^1^Department of Psychology, Arizona State University, TempeAZ, USA; ^2^Department of Psychology, University of Wisconsin-Madison, MadisonWI, USA; ^3^Simpson College, IndianolaIA, USA

**Keywords:** infantile amnesia, childhood amnesia, aging, memory, locomotion, embodiment

## Abstract

At least since the late nineteenth century, researchers have sought an explanation for infantile amnesia (IA)—the lack of autobiographical memories dating from early childhood—and childhood amnesia (CA), faster forgetting of events up until the age of about seven. Evidence suggests that IA occurs across altricial species, and a number of studies using animal models have converged on the hypothesis that maturation of the hippocampus is an important factor. But why does the hippocampus mature at one time and not another, and how does that maturation relate to memory? Our hypothesis is rooted in theories of embodied cognition, and it provides an explanation both for hippocampal development and the end of IA. Specifically, the onset of locomotion prompts the alignment of hippocampal place cells and grid cells to the environment, which in turn facilitates the ontogeny of long-term episodic memory and the end of IA. That is, because the animal can now reliably discriminate locations, location becomes a stable cue for memories. Furthermore, as the mode of human locomotion shifts from crawling to walking, there is an additional shift in the alignment of the hippocampus that marks the beginning of adult-like episodic memory and the end of CA. Finally, given a reduction in self-locomotion and exploration with aging, the hypothesis suggests a partial explanation for cognitive decline with aging.

There is no lack of theories to account for IA and CA. Bauer (2015) reviews these theories and explicates her Complementary Processes theory. We take that theory as a starting point, but add what we think is a crucial component based on embodiment, namely, the onset of locomotion. We will argue that it is the onset of locomotion, first crawling and then walking, that provides the conditions for hippocampal place cell and grid cell development, and that in turn allows for long-term storage and retrieval of autobiographical memories.

## Bauer’s Complementary Processes Theory

The first of the complementary processes (or perhaps better, complementary explanations) is that infants lack some of the abilities needed to form episodic memories. For example, Newcombe (e.g., Balcomb et al., 2011; Newcombe et al., 2014) propose that infants cannot bind elements of representations (e.g., time, place, actors, etc.) into events. Howe and Courage (1997) propose that infants lack a self-concept, and hence cannot relate memories to the self. Nelson and Fivush (2004) elaborate on this idea by including, for example, development of processes that control conversation and narrative structure, as necessary for the full expression of autobiographical memory. Whereas these ideas all have some support, we do not view the Howe and Courage and Nelson and Fivush accounts as central to IA and CA for the following reason: not just humans, but all altricial species show a form of IA (Arnold and Spear, 1997). On the assumption that IA across species is not accidental, we need an explanation that does not focus on human-centric concepts such as language and the self.

The second complementary explanation in Bauer’s theory is that even the episodic memories that infants and toddlers manage to form are exceptionally vulnerable to forgetting during early years. Bauer, and others, attribute changes in vulnerability to forgetting to maturation of the hippocampus. The hippocampus is a limbic structure located deep within the temporal lobe. It plays a crucial role in binding the spatial and temporal characteristics of long-term episodic memories (e.g., Moscovitch et al., 2006) by coding (or binding) environmental features as an organism moves through time and space. There is little doubt that the hippocampus plays a central role in episodic memory, and there is little doubt that it changes over the course of development. But why does the hippocampus mature at one time and not another, and how does that maturation relate to memory? We propose an embodied answer to these questions.

## Contribution of Embodiment

### The Hippocampus and Memory

The primary purpose of the hippocampus seems to be spatial navigation. Place cells code particular locations (e.g., O’Keefe and Nadel, 1978); grid cells code spatial relations amongst those locations (e.g., Hafting et al., 2005; Castro and Aguiar, 2014); and head direction cells code the current direction of movement (e.g., Taube, 1998). Reciprocal connections between the hippocampus and sensorimotor and emotional cortices, along with Hebbian learning, then produce the ingredients for episodic memory. That is, activity in visual and motor areas (for example) are bound together via the hippocampus which codes the location of the event, resulting in a specific memory of an event at a specific time and place. As the animal changes location, other place and grid cells can be used to bind new memories that occur while at the new location. Thus, the place and grid cells (a) bind together components of events, (b) protect similar events occurring in different locations from catastrophic interference (because of the association with different places), and (c) provide a location-based retrieval cue, much as with the mnemonic method of loci.

However, consider the following: Why would an animal that cannot engage in self-locomotion need to track location in the environment? The immature altricial animal is completely dependent on caretakers for food, warmth, and transportation. The animal does not explore, and hence there is no need to track novel locations. The animal does not leave the caretaker, so there is no need to track the location of the home environment. Even if the animal were separated from its caretaker, because it does not yet self-locomote, it could not get back home on its own. Thus, there is no need for evolution to have engineered an operating location-tracking mechanism for the animal that cannot self-locomote.

### Tuning the Hippocampus to the Environment

Furthermore, consider how location-tracking mechanisms, such as place cells and grid cells, might become tuned to their environment. One hypothesis is that the tuning comes about through the consistent correlation amongst several cues including optic flow, head direction, and proprioception from self-generated movements. For example, optic flow correlated with proprioception (a) defines for the animal how speed of locomotion translates into change of location, and (b) that correlation comes to represent what is expected of a stable world (Campos et al., 1992; Dahl et al., 2013). To help understand these claims, consider what happens when optic flow and proprioception are decoupled. One way to do that is by walking on a treadmill. While on the treadmill, the brain relearns the correlation so that now proprioception is paired with little flow. One consequence is that when stepping off the treadmill there is a brief period of disorientation during which the visual world appears to move too fast when one begins to walk (Pelah and Barlow, 1996). A second example is when sitting in a car at a red light and the car next to you starts to move, thus generating peripheral optic flow. A typical response is to slam on the brake because the peripheral optic flow is interpreted as self-movement.

Consider now the pre-locomotor infant who is carried from location to location. Because the infant is not constrained to keep her head pointed in the direction of travel, optic flow, head direction, and proprioception are for the most part uncorrelated. Thus, there can be no tuning of the place and grid cells to the local environment. Again, a commonplace experience may help to elucidate the idea. Imagine two people navigating to a new location, for example a driver and a passenger (or two tourists walking together in a new city, where one has a map and the other follows). In this situation, the driver is forced to keep her gaze focused in front, creating optic flow correlated with speed of locomotion, whereas the passenger engages in conversation (while looking at the driver), or looks around at the sights. Thus, for the passenger, there is an inconsistent correlation between optic flow, direction of movement, and speed of locomotion (although effects of active versus passive control must also be considered, as in Bakdash et al., 2008). The result is that the driver learns the route, but the passenger does not.

In summary, the pre-locomotor infant does not have the opportunity to tune his hippocampal place and grid cells to the environment. Consequently, the system remains immature and an unreliable contributor to memory. That is, it is difficult for the infant to associate events with a particular place because places are not strongly distinguished by spatial codes. Furthermore, it is difficult to keep track of separate but similar events experienced in different locations (because locations are not strongly differentiated), and this lack of differentiation results in mutual interference and forgetting of the similar events. Note that this hypothesis regarding the pre-locomotor infant corresponds to the first of Bauer’s complementary explanations: the infant is unable to form stable episodic memories. However, rather than suggesting a cause particular to humans, such as a poorly developed conception of the self or poor language skills, we offer a mechanism that might be found in every altricial species, namely, a lack of opportunity to tune place and grid cells to the environment, and hence a reduced capability to code the locations of experienced events.

Now consider the situation as the infant begins to crawl. She must keep her head pointed toward her goal, and that generates correlations between optic flow, head direction, and proprioception. We propose that these stable correlations provide the opportunities required to establish a stable system of grid cells linking places. Furthermore, with the advent of crawling, the infant can explore components of her environment (e.g., where the grassy area is relative to the tree; where the kitchen is relative to the TV) and differentiate them in terms of their spatial locations coded through linked grid and place cells. This system then provides the substrate for coding memories of events in distinct locations and keeping track of separate but similar events experienced in different locations. With the establishment of spatial location coding, long-term episodic memory becomes possible. This component of our hypothesis corresponds to the second of Bauer’s complementary explanations: vulnerability to forgetting declines over development.

### Re-tuning the Hippocampus

But why is there also CA, that is, continued rapid forgetting even of events experienced by the self-locomoting infant and toddler? One possibility is that it just takes a lot of experience in crawling and exploration to establish a hippocampus with sufficient organization of locations to work with adult-like efficiency. Consider, however, the following: at some point, the infant transitions from crawling to walking. With that transition, there is a new pattern of correlations between head direction, optic flow, and proprioception. The new pattern comes about because the angle between the head and ground (which contributes to optic flow) is changed, the pattern of muscles that produce locomotion and proprioception changes, and the relation between proprioception and optic flow (e.g., speed of movement and speed of flow) changes (Adolph and Tamis-LeMonda, 2014). This changing pattern of information forces a new organization of grid cells coding the relations between self-movement and change in location. With that re-organization, the hard-won episodic memories encoded during the crawling period become less accessible, that is, they are forgotten at a rate faster than that of an adult. Furthermore, it is not until walking and associated exploration are well-established that an adult-like hippocampal system can be formed, binding the elements of longer-lasting episodic memories.

Components of this hypothesis are not without precedent; Rovee-Collier (1996) anticipated at least two parts of the hypothesis. First, she noted that what infants learn matches their needs for their current ecological niche. For example, newborns learn appetitive responses, but not defensive ones. She wrote, “…newborns are motorically incapable of escaping or avoiding aversive events signaled by cues in the environment…Instead, the newborn’s first line of defense is to cry…Not surprisingly, most of the successful examples of aversive conditioning in infants have been obtained after the age of independent locomotion…” (p. 388). Similarly, we propose that infants are unlikely to develop functioning place and grid cells before the onset of “independent locomotion.” Second, she confirms both experimentally and theoretically the relation between self-locomotion and learning spatial relations, “…once infants are able to self-locomote…their definition of context seems to change. Before this time, they know what happens in what place, but they do not know how to get there. Once they can get there without being carried, however, they acquire spatial relations along with their new navigational skills and begin to construct a cognitive map…This is particularly important because *once these different places become related to each other, the memories of events that transpired in those places also become associated with each other*” (p. 393, emphasis in the original). Nonetheless, Rovee-Collier offers a different explanation for IA and CA.

## Evidence Specific to the Embodied Hypothesis

The embodied hypothesis is broadly consistent with the basic facts of IA and CA. Furthermore, by noting the developmental transitions between crawling and walking, the hypothesis provides a simpler explanation of continued weak episodic memory than many other hypotheses. In addition, there are reports that provide evidence consistent with specifics of the hypothesis. What follows is not an exhaustive review of the literature, but a sampling to illustrate some of the highlights.

Herbert et al. (2007) found that 9-months-old infants who had begun crawling were able to successfully perform a deferred imitation task when asked to perform the task in a novel environment with a similar but unique object. Same aged, but immobile infants, were not as successful. As Rovee-Collier (1996) suggests, the mobile infants are better able to associate places with one another and thus retrieve events learned in one environment while located in a different environment.

The literature also documents important changes in cognition that accompany the acquisition of self-locomotion (many are reviewed in Campos et al., 2000). For example, Campos et al. (1992) demonstrate how fear of the visual cliff is dependent on self-locomotion. They explain that it is only after the onset of self-locomotion that the relation between optic flow and proprioception comes to define a stable world. Then, when that relation is disrupted by the unusual optic flow near a visual cliff, the infant recognizes a new (and potentially dangerous) type of environment.

Particularly pertinent to our hypothesis are the findings of (Adolph, 1997; Adolph and Tamis-LeMonda, 2014). She documents changes in cognition associated with the transition from crawling to walking. For example, after many weeks of crawling, infants are able to discriminate between slopes that are safe for crawling and those that are not safe. When the infant begins to walk, however, that discrimination is lost, and the toddler must again learn to discriminate between these slopes. Adolph’s discussion of this result (e.g., pp. 66–68; 125–126) is consistent with changes in optic flow and proprioception associated with the transition from crawling to walking.

Bauer et al. (2012) documents how 4-years-old children (compared to 6- and 8-years-old) are not much better than chance in remembering the locations of events. Interestingly, Riggins et al. (2015) uncovered an association between episodic memory and hippocampal volume for 6-years-old, but not for 4-years-old.

Perhaps most important for our hypothesis, the relation between grid cell firing and self-locomotion is now clearly established (at least for rats). Winter et al. (2015) had rats explore an environment while recording from hippocampal grid cells. In the active condition, the rats explored the environment through self-locomotion. In the passive condition, the animal was pulled through the environment in a clear cart. There were several important findings. First, grid cell firing was closely associated with environmental location of the animal in the active condition, but was severely degraded or missing in the passive condition. That is, much like passengers in a car, the rats in the passive condition were not tracking how they got from one location to the next. Second, the firing of head direction cells (that provide the brain with a heading) was not disrupted in the passive condition, but third, the correlation between oscillations in the theta band (6–10 Hz) and speed of locomotion was disrupted. The second and third findings help to explain the first finding of disorganized grid cell firing in the passive condition. Namely, while in the cart, the animal’s head cues the direction of travel. However, the speed of travel, normally associated with movement of and proprioception from the animal’s own legs, translated into theta rhythm, is disrupted. Thus, although there is firing in the theta range, it is dissociated from speed of movement (now provided by the cart instead of the animal’s legs), and hence there is a disruption in signaling of when grid cell boundaries are crossed.

## How can this Hypothesis be Tested?

Some predictions can be tested with animal models. For example, weanling rats can be allowed to explore an open field environment that does or does not contain differentiating features. The animals exposed to the differentiated environment should form a more differentiated grid cell system. Then, episodic memory can be assessed by placing food rewards in specific locations in the radial arm maze where the animal can eat the reward. We predict that rats with differentiated locomotive experience will be less likely to return to the empty locations compared to rats reared in an undifferentiated environment.

Other hypotheses can be tested with human infants (several of these ideas are identical to those in Campos et al., 1992). For example, there should be a correlation between age of onset of crawling (and walking) and performance in episodic memory tasks. A correlation may also be found across cultures that differ in child-rearing practices that encourage or discourage self-locomotion. Similarly, children who have been injured so that self-locomotion is delayed should show a delay in onset of episodic memory.

Experimental tests are also possible. Consider the following test of a counterintuitive prediction (see **Table 1**). In Phase 1 of the experiment, infants who are still crawling are taught a task (Task 1). Phase 2 occurs when the infant takes her first steps, but while she is still predominately a crawler. In Phase 2, the infant is tested on Task 1 and learns a new task, Task 2. Phase 3 is scheduled so that the interval between Phases 2 and 3 is the same as the interval between Phases 1 and 2. In Phase 3, the toddler is tested on Task 2. We predict that the young infant will do relatively well on the test of Task 1 (at Phase 2) because she learned it while having a “crawling” hippocampus and was tested while having a “crawling” hippocampus. However, when the older child is tested on Task 2 (Phase 3), she should do less well because Task 2 was learned while the child had a “crawling” hippocampus, but is tested while she has a “walking” hippocampus.

**Table 1:** An experimental test of the hypothesis.

	Phase of experiment		
	
	(1) Child is crawling	(2) First steps	(3) Child is walking
Hypothetical mode of tuning hippocampus to environment	Crawling	Primarily crawling	Walking
Task learned	Task 1	Task 2	
Task tested		Task 1	Task 2


## Discussion

Although we developed this hypothesis to address IA and CA, it also suggests a novel explanation for some forms of cognitive decline with aging. That is, as the body ages, there may be less self-locomotion and less active exploration. In this case, hippocampal place and grid cell firing may become dissociated from the environment, with a resultant decrease in episodic memory. Prevention of detuning may result from following the common advice to remain active while aging. Furthermore, this extension of the hypothesis suggests an explanation for results reported by Anderson-Hanley et al. (2012). They tested a sample of older adults (average age of 78.8 years) randomly assigned to ride a stationary bicycle (for 3 months) or the same bicycle combined with a virtual reality tour simulating movement through the environment. Riding the stationary bicycle with the virtual tour provided significantly more buffering against mild cognitive impairment than riding the stationary bicycle alone. Furthermore, this buffering effect was associated with a greater increase in BDNF, brain-derived neurotrophic growth factor.

In summary, we have developed a novel explanation for IA and CA. In brief, when an animal cannot self-locomote, there is no opportunity for hippocampal place and grid cells to be tuned to the environment. Consequently, episodic memories (which require memory for place) are difficult to establish. The onset of self-locomotion provides the opportunity for tuning place and grid cells to the environment, and thus the onset of episodic memory. In humans, however, the change in mode of locomotion from crawling to walking rewires the hippocampal cells so that memories created in the crawling phase are less available, thus providing at least a partial explanation for IA and a more extended CA. As the animal continues to explore wider and more diverse areas, a greater number of hippocampal cells can become tuned to the environment, thus establishing a mature basis for coding the locations of remembered events. Unlike some other explanations of IA, this hypothesis provides an explanation that holds across altricial species.

## Author Contributions

AG contributed to developing the hypothesis and writing the manuscript. JH contributed to developing the hypothesis and writing the manuscript.

## Conflict of Interest Statement

The authors declare that the research was conducted in the absence of any commercial or financial relationships that could be construed as a potential conflict of interest.
